# Evolution of Genome Size and Complexity in *Pinus*

**DOI:** 10.1371/journal.pone.0004332

**Published:** 2009-02-05

**Authors:** Alison M. Morse, Daniel G. Peterson, M. Nurul Islam-Faridi, Katherine E. Smith, Zenaida Magbanua, Saul A. Garcia, Thomas L. Kubisiak, Henry V. Amerson, John E. Carlson, C. Dana Nelson, John M. Davis

**Affiliations:** 1 School of Forest Resources and Conservation, University of Florida, Gainesville, Florida, United States of America; 2 Department of Plant and Soil Sciences, Mississippi State University, Mississippi State, Mississippi, United States of America; 3 Southern Institute of Forest Genetics, USDA Forest Service Southern Research Station, Saucier, Mississippi, United States of America; 4 Department of Forestry and Environmental Resources, North Carolina State University, Raleigh, North Carolina, United States of America; 5 School of Forest Resources, The Pennsylvania State University, University Park, Pennsylvania, United States of America; Louisiana State University, United States of America

## Abstract

**Background:**

Genome evolution in the gymnosperm lineage of seed plants has given rise to many of the most complex and largest plant genomes, however the elements involved are poorly understood.

**Methodology/Principal Findings:**

*Gymny* is a previously undescribed retrotransposon family in *Pinus* that is related to *Athila* elements in *Arabidopsis*. *Gymny* elements are dispersed throughout the modern *Pinus* genome and occupy a physical space at least the size of the *Arabidopsis thaliana* genome. In contrast to previously described retroelements in *Pinus*, the *Gymny* family was amplified or introduced after the divergence of pine and spruce (*Picea*). If retrotransposon expansions are responsible for genome size differences within the Pinaceae, as they are in angiosperms, then they have yet to be identified. In contrast, molecular divergence of *Gymny* retrotransposons together with other families of retrotransposons can account for the large genome complexity of pines along with protein-coding genic DNA, as revealed by massively parallel DNA sequence analysis of Cot fractionated genomic DNA.

**Conclusions/Significance:**

Most of the enormous genome complexity of pines can be explained by divergence of retrotransposons, however the elements responsible for genome size variation are yet to be identified. Genomic resources for *Pinus* including those reported here should assist in further defining whether and how the roles of retrotransposons differ in the evolution of angiosperm and gymnosperm genomes.

## Introduction

Gymnosperms (conifers, cycads, gnetophytes and ginkgo) have among the most complex and largest genomes of any living organisms. Pine trees, conifers belonging to the genus *Pinus*, are excellent subjects for dissecting processes involved in genome evolution for several reasons. Evolutionary forces have acted on pine genomes since they diverged from the most closely related genus *Picea* (spruces) 87 to 193 MYA [1]. The genus has a rich history of phylogenetic analysis so the relationships among the approximately 120 extant species in the genus are well understood [2], [3]. Genetic conservation has been implemented for many different pine species, organized by cooperative programs headquartered at public institutions [4], [5], which enables researcher access to germplasm. Pines have genome sizes ranging between 18,000 and 40,000 Mbp (1C content) and precise measures of genome size have enabled direct comparisons of 1C nuclear DNA content among many species [1], [6], [7]. In contrast to large angiosperm genomes (most prominently maize) where gene duplications, diverse chromosome numbers and genome size variation among related species indicate historical polyploidization complemented by periods of retrotransposon expansion [8], [9], all extant members of the genus *Pinus* are diploid with 2*n* = 24 chromosomes. Induced polyploids in *Pinus* show poor survival and growth and interspecific hybridization does not increase the genome size of *Pinus* hybrid offspring to levels above either parent [10]. Therefore, periods of retrotransposon expansion and not polyploidy may be of primary importance in explaining genome size variation within *Pinus*. Pines are well-represented in paleoflora [2], [11], which calibrates dates of divergence among monophyletic groups [12], and this information could be used to identify intervals during which retrotransposons have been introduced or amplified.

Retrotransposons, mobile genetic elements propagated via a “copy and paste” mechanism involving an RNA intermediate, comprise the majority of noncoding DNA and have greatly expanded the genomes of many angiosperms [13]. Of the five major orders of retrotransposons, the long terminal repeat (LTR) order predominates in plant genomes [14]. LTR retrotransposon regions and domains are well-defined [15], [16] and their relative position and sequence distinguishes *Ty1/Copia*-like or *Ty3/Gypsy*-like elements. Nonautonomous elements can still transpose but this depends on enzymes encoded elsewhere in the genome [17]. Periods of retrotransposon activity have punctuated the evolution of modern plant genomes [14], [18], [19]. These expansions may accompany genomic or environmental stress, potentially establishing the heritable variation on which selection can act to form new species [20]–[22]. Of the few LTR retrotransposons that have been identified in *Pinus* spp., all are also present outside of the genus [23]–[26]. However, the identification of a *Gypsy* element apparently unique to *Picea* [23] implies there are taxon-specific retroelements whose activity could be associated with speciation.

Sequence complexity describes all the novel sequence information in a genome [reviewed in 27] and can be expressed as a proportion of genome size or in base pairs. Genome complexity can be estimated by Cot analysis, which is a technically challenging method used in 86 published manuscripts prior to 1990 [27], but not in common use after the availability of massively parallel sequencing approaches. Cot analysis can provide valuable information for genomes that are not yet sequenced, as it enables separation of non-redundant (low copy, protein-coding genes) from redundant (high copy, repetitive including retrotransposon) sequences. Genome complexity in angiosperms varies from 13% (*Allium cepa*) to 77% (*Solanum lycopersicum*) with a mean of 39%. Expressed in base pairs, genome complexity values for well-studied diploid angiosperms are 82.6 Mb (*Arabidopsis thaliana*), 290 Mb (*Sorghum bicolor*), 735 Mb (*Solanum lycopersicum*) and 955 Mb (*Zea mays*) [28], [29]. In the only report in which gymnosperm genome complexity estimates were compared, values expressed as a proportion of genome size are similar to that of angiosperms and range from 24% (mean for three *Pinus* spp.) to 71% (for *Picea glauca*) [30]. Expressed in base pairs, however, it becomes clear that conifer genome complexity is enormous compared to typical diploid angiosperms; 2,890 Mb (*Pinus banksiana*), 5,160 Mb (*Pinus resinosa*), 5,740 Mb (*Picea glauca*) and 7,820 Mb (*Pinus lambertiana*) [27]. Cot-based fractionation has been coupled with high-throughput sequencing to show enrichment of genic DNA in maize [31]–[33], however this approach has not yet been reported for any gymnosperm.

In this manuscript we introduce *Pinus taeda* genomic resources including a BAC library and datasets from massively parallel sequencing of Cot-based fractionated DNA. A previously undescribed LTR retrotransposon family (*Gymny*) occupies a physical space at least as large as the entire *Arabidopsis thaliana* genome (157 Mbp, [34]) and appears specific to subgenus *Pinus*. Although most *Gymny* sequences are detected in the high copy fraction of the *Pinus* genome as expected, 18–19% are found in the low copy fraction along with protein-coding genes. Retrotransposon expansion followed by mutation of similarly taxon-specific families of retrotransposons could account for both the size and complexity of modern pine genomes. Public sequence datasets now available should encourage more studies to characterize the evolution of retrotransposons in the genomes of gymnosperms, which include many of the most ecologically, evolutionarily and economically important plant species on the planet.

## Results

### *Gymny* is related to *Athila* but dispersed in the genome

**Figure 1:**
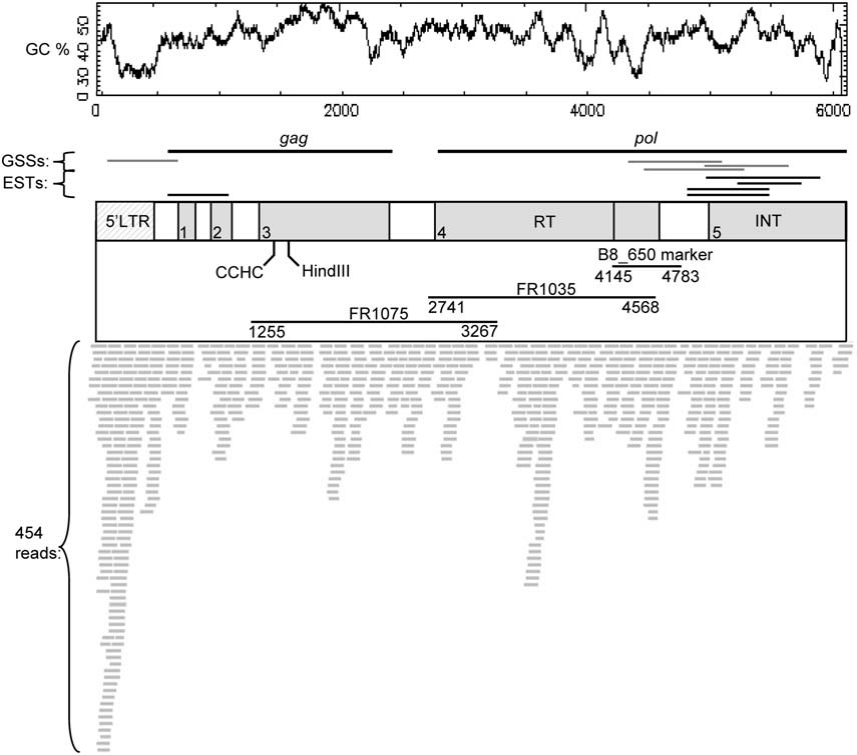
Organization of the RLG_*Gymny*_EU912388-1 retrotransposon. Percent G+C is shown above the *Gymny* schematic. Numbered ORFs are in gray with vertical lines indicating stop codons, putative 5′LTR as hatched box, PR protease, RT reverse transcriptase, INT integrase. ESTs, GSSs, and 454 reads are indicated as are the B8_650 marker, and the Southern probes Fr1075 and Fr1035.

Retrotransposon integration and divergence can introduce genetic polymorphisms that can be detected as randomly amplified polymorphic DNAs (RAPDs) [35]. Here we describe the identification of the reference *Gymny* element (RLG_*Gymny*_EU912388-1), starting from the sequence of a RAPD marker linked to the fusiform rust resistance locus *Fr1* [36], beginning from the 650 bp sequence of the RAPD marker B8_650. The final sequence was annotated (File S1) and aligned with reads from massively parallel sequencing of *P. taeda* genomic DNA, GSS and ESTs (Figure 1; Table 1). The consensus sequence of the largest contig (assembled *in silico*) that aligns with RLG_*Gymny*_EU912388-1 is >90% identical to the query, which indicates the reference is representative of the *Gymny* family in *P. taeda*.

**Table 1:** *Gymny* in *Pinus taeda* sequence databases (GenBank).

Source	Accession	Name	Lib^a^	Position (bp)^b^
dbEST	DR101053.1 (3′), DR101125.1 (5′)	STRR1_70_F01	PC	*int* 4959–5893
dbEST	BQ290602.1	NXRV047_D04_F NXRV	XR	*int* 5199–5761
dbEST	DT628148.1	EST1156897	SE	*int* 4811–5448
dbEST	DN611113.1	EST964163	SE	*int* 4811–5449
dbEST	BQ655822.1	NXRV099_G06_F NXRV	XR	*gag* 603–1114
dbGSS	ET182012.1	PT_7Ga_B01_00001_G22_r	TG	4314–5188
dbGSS	CZ896063.1	226_2_12341072_5489_37963_058	MU	4982–5641
dbGSS	ET182110.1	PT_7Ga_B01_00002_B23_r	TG	4468–5271
dbGSS	CZ895334.1	upta001f001a09f1	MU	79–681

a Libraries: PC, pitch canker resistant stem; XR, xylem root wood vertical; SE, subtracted pine embryo; TG, pine total genomic DNA; MU, pine methylation unfiltered library.

b As defined in Figure 1.

**Figure 2:**
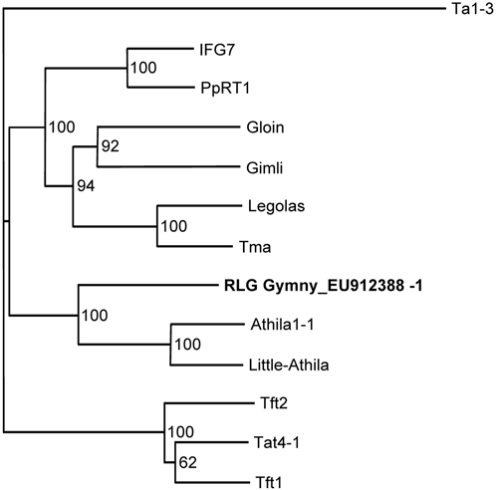
RLG_*Gymny*_EU912388-1 is related to Arabidopsis *Athila*-like retrotransposons. Relatedness tree generated from alignment of RT sequences in Figure S1 and the RT domain from the Arabidopsis *Ta1-3 Copia*-like retrotransposon (Accession number X13291; [38]). Bootstrap percent values based on 10,000 replications.

RT polymerase domains are generally the most conserved regions of retrotransposons [37]. The order of the predicted coding sequences of RLG_*Gymny*_EU912388-1 and similarity of the RT domain place it in the *Gypsy* superfamily (Figure S1). A relatedness tree (Figure 2) was constructed using RT domains from selected *Gypsy* elements and from *Ta1-3*, a *Copia* retrotransposon from *Arabidopsis* [38]. RLG_*Gymny*_EU912388-1 forms a well-supported clade with the *Athila* group of retroelements and is distinct from previously characterized pine *Gypsy* retrotransposons (IFG7 and PpRT1) and *Ta1-3*.

**Figure 3:**
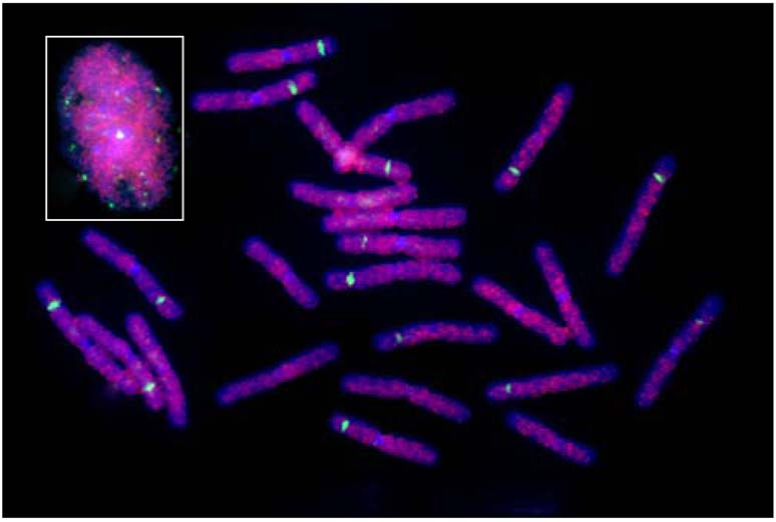
FISH showing the physical distribution of *Gymny* in somatic chromosome spread of *Pinus taeda*. RLG_*Gymny*_EU912388-1 probes Fr1035 and Fr1075 were detected with Cy3 streptavidin (red) and 18S–28S rDNA was detected with FITC (green). Inset shows FISH to interphase nucleus.

*Athila* elements are clustered in pericentromeric regions of *Arabidopsis* based on FISH and genomic data mining [39], [40]. *Gymny* showed no consistent localization with centromeric (primary constrictions in the chromosomes), pericentromeric or telomeric regions (Figure 3).

### *Gymny* family size is at least as large as the *Arabidopsis* genome

**Figure 4:**
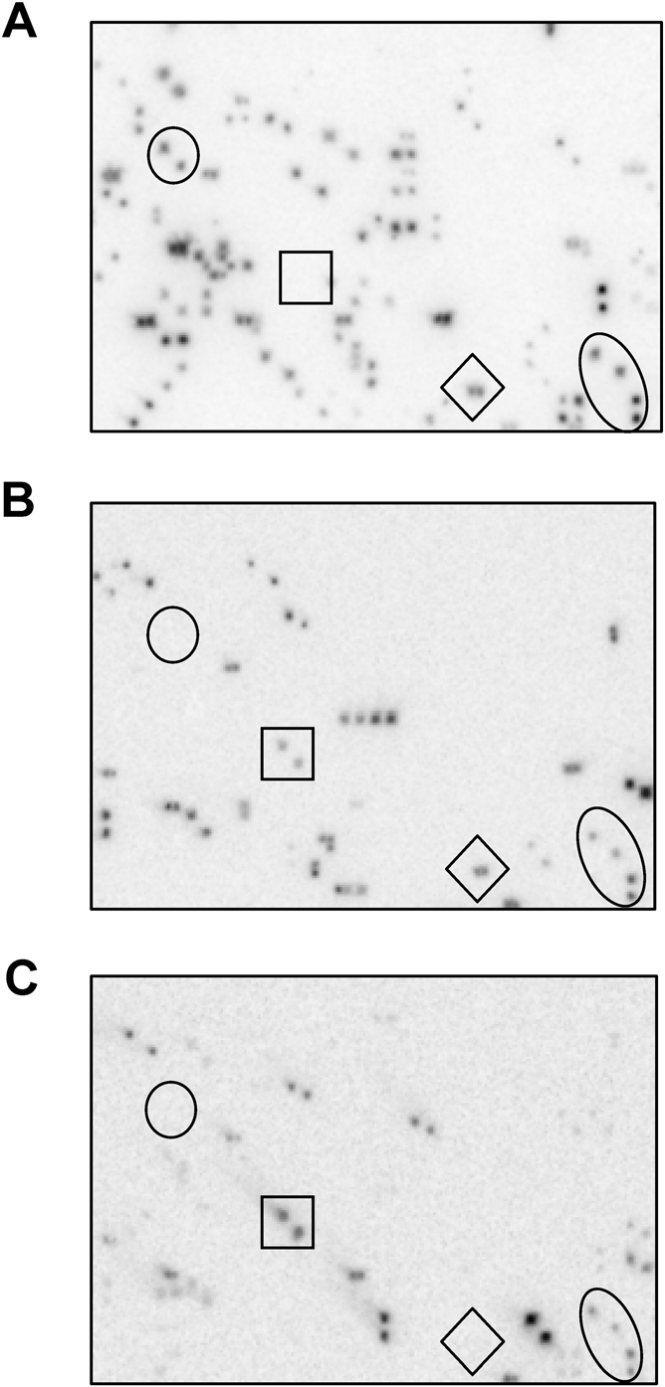
The same region of three identical BAC macroarrays hybridized with (A) probe P1; (B) probe P2; and (C) probe P3. Clones are spotted in duplicate so positive signal is a closely situated pair of spots. BACs within circles, squares, diamonds and ovals show differential hybridization among probes. While most symbols highlight a single BAC (i.e. a pair of spots), the oval highlights two BACs that hybridized to all three overgo probes.

To quantify the contribution of *Gymny* to genome size, we screened BACs with overgo probes derived from three different regions of the reference element. Of 18,432 BAC clones screened, 3.1% exhibited hybridization to one or more of the three probes (Table 2). If most copies of *Gymny* possess intact LTRs and internal regions with sequences similar to RLG_*Gymny*_EU912388-1, then most positive BACs would show hybridization to all three probes. However, the probes hybridized to partially overlapping subsets of BACs (Figure 4). Only 14.0% of positive clones showed co-hybridization with all three probes, whereas almost half (49%) of the positive BACs showed hybridization solely to the LTR (P1) probe, suggesting the presence of non-autonomous derivatives with intact LTRs but lacking some or all of the internal coding regions. Apparently *Gymny* derivatives are much more common than reference-like elements in the *P. taeda* genome.

**Table 2:** Co-hybridization of overgo probes on BAC macroarrays.

Probes	# Positive^a^	% Positive^b^	% of BACs^a^
P1,P2,P3	19	14.0	0.62
P1,P2	7	5.1	0.23
P1,P3	10	7.4	0.33
P2,P3	4	2.9	0.13
P1	67	49.3	2.18
P2	14	10.3	0.46
P3	15	11.0	0.49

a In macroarray section of 3072 BAC clones.

b Of 136 total hybridizing BAC clones.

So, how much DNA does the *Gymny* family contribute to the genome? Densitometric analysis of the macroarrays as per Peterson *et al.* [29] suggests the three overgos are found in 105,579, 88,203 and 42,569 copies per haploid genome, respectively. Given that LTR retrotransposons contain two LTR domains, the observed copy number ratios of 1.2 to 1 and 2.5 to 1 for P1 compared to P2 and P3, respectively, indicates that the LTR domains are not over-represented compared to the internal domains. Thus, the interrupted pattern of overgo hybridization may have arisen from element disruption rather than recombination. Each analyzed section of the macroarray contained 3072 BAC clones and represents 273,408,000 bp of pine DNA or 1.26% of the *Pinus taeda* genome (21.7 Gb, [41]). If we assume that the 0.62% of BAC clones showing hybridization to all three overgos (Table 2) each contain one copy of an element similar in structure to RLG_*Gymny*_EU912388-1, then the amount of DNA in RLG_*Gymny*_EU912388-1-like elements in the pine genome can be estimated as [(0.0062×273,408,000 bp)÷0.0126] = 134,534,095 bp or ∼135 Mb. We estimate copy number of elements similar to the reference by noting that RLG_*Gymny*_EU912388-1 is 6,113 bp in length but lacks an intact 3′ end. If we round the size of the element up to 6200 bp, then the pine genome may contain about (134,534,095 bp÷6200 bp) = 21,699 copies of elements similar in structure to RLG_*Gymny*_EU912388-1. An independent estimate of copy number (14,138) was obtained from the hit frequency in the 454 sequence dataset from genomic DNA (File S1). Our estimate that *Gymny* reference-like elements occupy ∼135 Mb of the pine genome does not include *Gymny* derivatives, which are far more abundant (Table 2).

### *Gymny* elements are found in low and high copy genomic fractions

To quantify the contribution of *Gymny* to genome complexity, we performed Cot-based fractionation of genomic DNA, carried out massively parallel DNA sequencing on the highly repetitive (HR), moderately repetitive (MR), single/low-copy (SL) and theoretical single-copy (TS) fractions, trimmed the datasets for quality and length, queried the datasets with RLG_*Gymny*_EU912388-1 and retrieved hits with bit scores >40 (Table 3). The MR fraction had the greatest proportion of reads with hits (0.67%), followed by HR (0.64%), SL (0.24%) and TS (0.18%). As expected, the random genomic (RG) dataset produced an intermediate value (0.40%). Results using a second analytical approach in which the total (unfiltered) datasets were each assembled into contigs, queried and hits retrieved based on E-value <10^−4^ detected higher frequencies of hits in each fraction (Table 3), however both approaches revealed similar proportions of *Gymny* elements in the genomic fractions relative to one another (Pearson's correlation, r = 0.97).

**Table 3:** *Gymny* and *WRKY* distribution in *Pinus taeda* genomic fractions.

Cot^a^	Total Bases	Trimmed^b^ Reads	*Gymny* c		Total Contigs	*Gymny* d		*WRKY* e Hits
			Hits	%		Hits	%	
RG	28,039,433	275,038	1,111	0.40	28,855	139	0.48	3 (9)
HR	28,047,400	216,921	1,397	0.64	22,163	197	0.89	0 (1)
MR	26,156,228	206,402	1,377	0.67	16,702	170	1.02	0 (1)
SL	20,235,555	102,708	245	0.24	14,681	50	0.34	3 (5)
TS	31,509,545	215,387	390	0.18	19,969	30	0.15	5 (18)

a Fractions: RG, random genomic; HR, highly repetitive; MR, moderately repetitive; SL, single/low copy; TS, theoretical single-copy.

b Low quality score and length reads removed, see Methods.

c On reads, bit score >40.

d On contigs, E value <10^−4^.

e On reads, unique hits bit score >50 (total hits bit score >40).

We then calculated the proportion of *Gymny* elements that contribute to the high copy combined fraction (“low complexity” or HR+MR) relative to the low copy combined fraction (“high complexity” or SL+TS) of the genome. For example, the proportion of sequences in the low copy combined fraction using the first approach (query of trimmed datasets and retrieval of hits with bit score >40) was [(245+390) / 3409] = 0.19. Both approaches generated similar estimates of the proportion of *Gymny* hits in high copy (81% and 82%, respectively) relative to low copy combined fractions (19% and 18%, respectively). While our hit frequencies may have overestimated the proportion of retrotransposon sequences in the low copy combined fraction (since the complexity of the *Pinus* genome is about 24%, whereas the proportion of sequences in SL+TS is 43% of the overall dataset), it is more likely that we have underestimated the true value. This is because we cannot detect retrotransposon sequences that have mutated so as to be undetected by BLAST query. These mutation events may reflect accumulation of point mutations, or occurrence of sites where retrotransposons insert within preexisting retrotransposons to create interrupted sequences of retroelements [42] – such that alignments do not exceed minimum bit score thresholds.

The accumulation of retrotransposon family derivatives has clearly enriched the complexity of the modern *Pinus* genome. In addition to *Gymny* (Table 3), we detected 15% of sequences from the pine *Copia* element TPE1 (GenBank accession Z50750) in the low copy combined fraction (data not shown).

To confirm the technical robustness of the genomic DNA fractionation procedure, we queried each dataset with 26 EST contigs derived the *WRKY* family of plant-specific transcription factors [43]. The number of different reads in each dataset with a strong hit (bit score >50) on at least one query ranged from 5 (in TS) to 3 (in SL) to 0 (in HR and MR, respectively; Table 3). Some reads hit on multiple queries; the total number of hits with bit score >40 in each dataset ranged from 18 (in TS) to 5 (in SL) to 1 (in HR and MR, respectively). The single hits in HR and MR each aligned with an A/C-rich tract in *WRKY* contig 10761 with a bit score of 42, however A/C-rich subtelomeric repeat sequences are abundant in HR and MR (data not shown), implying similarity to the *WRKY* is spurious. The distribution of *WRKY* sequences among the HR, MR, SL and TS databases contrasts sharply with that of *Gymny* elements, and provides strong evidence that the genome fractionation was robust. The number of different reads in the random genomic database can be used to estimate copy number using the same approach as for *Gymny* elements. Three unique hits on the random genomic database, assuming *WRKY* coding sequences average 1500 nt in length, yield an estimate of 158 copies in the pine genome. While this estimate is imprecise due to limited sampling, this hit frequency would be expected for a gene family roughly double the size of the Arabidopsis *WRKY* family (N = 72, Plant Transcription Factor Database [43]).

### *Gymny* history is unlike previously described elements

**Figure 5:**
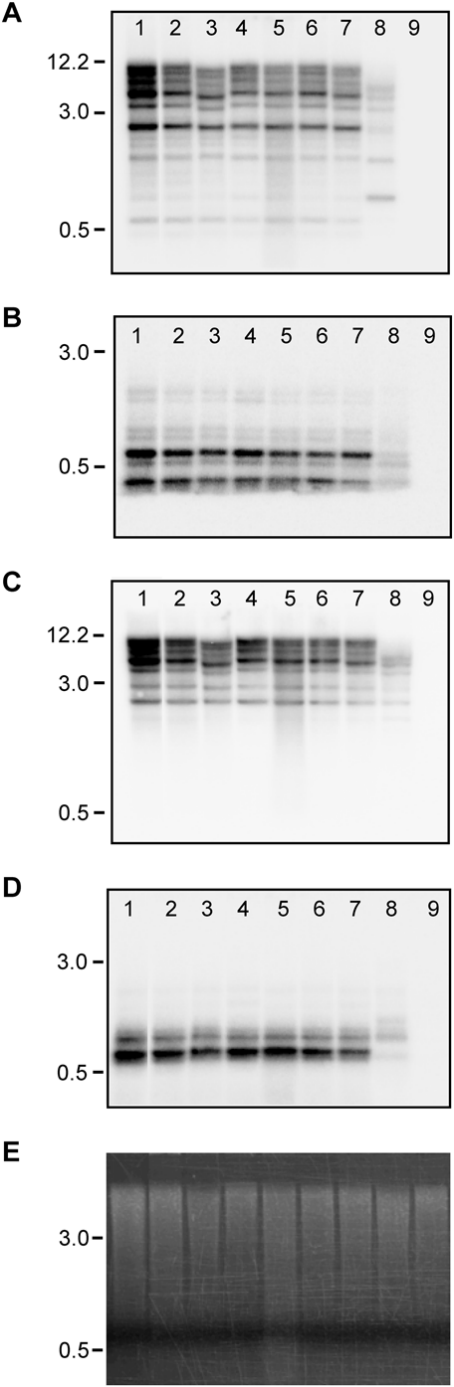
Southern blot analyses of selected *Pinus* species. The location of probes Fr1035 and Fr1075 in RLG_*Gymny*_EU912388-1 are indicated in Figure 1. (A, B) filters hybridized with Fr1075 probe; (C, D) filters hybridized with Fr1035 probe; (A, C) digested with *Hin*dIII; (B, D) digested with *Hae*III; (E) representative *Hin*dIII digested DNA stained with ethidium bromide. Lanes (1) *Pinus glabra*, (2) *P. taeda*, (3) *P. elliottii*, (4) *P. radiata*, (5) *P. echinata*, (6) *P. palustris*, (7) *P. virginiana*, (8) *P. pinea*, (9) *P. strobus*.

**Figure 6:**
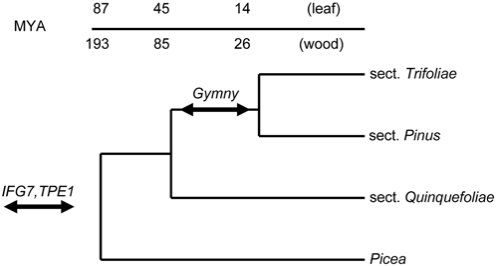
Monophyletic lineages within the genus *Pinus*, with hypothesized time frame for amplification or introduction of *Gymny* elements into the subgenus *Pinus* lineage relative to IFG7 and TPE1. The tree shown is derived from the analyses performed by Willyard et al. [12] where the dates for the nodes were selected to show the maximum possible range of values using either wood or leaf fossil calibrations, and either nuclear DNA or chloroplast DNA markers.

We tested presence and organization of *Gymny* in species representing a range of genome sizes [1], [44]–[46] across three monophyletic lineages within the genus *Pinus*, and other gymnosperms (Table 4) using probes derived from overlapping internal regions of RLG_*Gymny*_EU912388-1 (Southern probes Fr1035 and Fr1075, Figure 1). All seven pine species from subgenus *Pinus* section *Trifoliae* (Table 4) had equivalent hybridization patterns and signal intensities (Figure 5). *Pinus pinea* (subgenus *Pinus* section *Pinus*) also contains *Gymny*, but the family exhibits a distinct organization and decreased probe hybridization compared to pines in section *Trifoliae* (Figure 5, lane 8). This may reflect amplification of a structurally distinct *Gymny*-like element in the *Pinus pinea* ancestral line. *Gymny* was not detected in genomic DNA of *Pinus strobus* (subgenus *Strobus*), which implies its amplification or introduction after differentiation of the subgenera, but prior to differentiation of the two monophyletic lineages within subgenus *Pinus* (Figure 6), a time interval between 16–85 MYA depending on the dated fossils used for calibration and whether nuclear or plastid markers are used to date divergence [12]. Restriction of *Gymny* to *Pinus* was verified by Southern hybridization (negative results in conifers *Picea glauca*, *Picea mariana*, *Picea rubens*, *Tsuga canadensis*, *Abies fraseri*, *Ginkgo biloba*, and angiosperms *Populus trichocarpa*, *Arabidopsis thaliana*, *Sorghum bicolor*) and no *Gymny* hits to *Picea* spp. ESTs (N = 468,703). In contrast, IFG7 and TPE1 queries each generated multiple hits in both *Pinus* and *Picea* EST collections.

**Table 4:** Gymnosperms used in Southern blot analysis.

Species^a^	*Pinus* Subgenus	*Pinus* Section	*Pinus* Subsection	Source^b^
*Pinus echinata* Mill.	*Pinus*	*Trifoliae*	*Australes*	SIFG
*Pinus elliottii* Engelm. var. *elliottii* (LA-11)	*Pinus*	*Trifoliae*	*Australes*	SIFG
*Pinus glabra* Walt.	*Pinus*	*Trifoliae*	*Australes*	SIFG
*Pinus palustris* Mill. (3–356)	*Pinus*	*Trifoliae*	*Australes*	SIFG
*Pinus pinea* L.	*Pinus*	*Pinus*	*Pinaster*	SIFG
*Pinus radiata* D. Don	*Pinus*	*Trifoliae*	*Attenuautae*	Camcore
*Pinus taeda* L.	*Pinus*	*Trifoliae*	*Australes*	SIFG
*Pinus virginiana* Mill.	*Pinus*	*Trifoliae*	*Contortae*	SIFG
*Pinus strobus* L.	*Strobus*	*Quinquefoliae*	*Strobi*	NCSU
*Abies fraseri* (Pursh) Poir.	-	-	-	NCSU
*Ginkgo biloba* L.	-	-	-	NCSU
*Picea glauca* (Moench) Voss	-	-	-	PSU
*Picea mariana* (Mill.) Britton et al.	-	-	-	PSU
*Picea rubens* Sarg.	-	-	-	PSU
*Tsuga canadensis* (L.) Carr.	-	-	-	NCSU

a Open-pollinated seedlings of unknown or known maternal origin, except where noted.

b SIFG, Southern Institute of Forest Genetics, Saucier, MS; Camcore, North Carolina State University, Raleigh, NC (Dr. Gary Hodge); NCSU, Dr. John Frampton, North Carolina State University, Raleigh, NC; PSU, Dr. John Carlson, The Pennsylvania State University, University Park, PA.

## Discussion

### How and why did pine genomes become so complex?

The sequence complexities of three modern pine genomes constitute about 3,000 to 8,000 Mb, much larger than typical for diploid angiosperms [27]. Two competing but not mutually exclusive hypotheses can be proposed to explain these differences in genome complexity. Genic DNA may have increased in pines relative to angiosperms – gene families are larger [47] and unique cDNA-derived SAGE tags are more abundant [47], [48]. Alternatively, retrotransposon derivatives may have accumulated in the low copy fraction, thereby inflating it [49], [50]. Our findings support the retrotransposon derivative hypothesis. Based on frequency distributions of divergent members within retrotransposon families, similar processes are likely occurring in *Sorghum bicolor* [29] and *Oryza australiensis* [51]. If retrotransposons constitute the vast majority of the *Pinus taeda* genome, then the overall contribution of retrotransposon derivatives would be sufficient to explain most of its massive complexity.

The dispersed pattern of *Gymny* elements, shared with many other pine *Gypsy* elements and TPE1, is in contrast to the tendency of many *Gypsy*-family retrotransposons to cluster in centromeric and pericentromeric regions in most [52]–[55], but not all [56] angiosperm species. Like the *Copia* element TPE1 [24], most *Gypsy* elements were randomly dispersed across *Pinus* chromosomes, however one exceptional clone (Ppgy1) localized to centromeres [23]. This finding implies a potential impact of many retroelements, including *Gymny*, on the expression of neighboring genes. Transcribed retrotransposon derivatives could also account for novel SAGE tags [48] and appear to represent genic DNA [57]. BAC sequencing will help establish the spatial relationships among retroelements and neighboring genes as well as the relative timing of their activities [19].

### How and why did pine genomes become so large?

Retrotransposons have presumably contributed to the large size of modern gymnosperm genomes. The *Gymny* family is a recent addition to the *Pinus* genome, having been introduced or amplified as recently as 16 MYA. This stands in contrast to other described retrotransposons in *Pinus*, which predate the divergence of *Pinus* and *Picea* (at least 87 MYA). While retrotransposon expansion is a reasonable hypothesis for genome size evolution in pines, the retrotransposon families responsible have not yet been reported. We draw this conclusion because related species with distinct genome sizes have either similar retroelement copy numbers based on Southern hybridization intensities, or species with larger genomes have lower copy numbers (this work; [23]–[25]. However, a mere 10-fold expansion of a *Gymny*-sized family would be sufficient to explain the ∼1300 Mb of genome size variation within subsection *Australes* [1], [12]. Genome size variation could also be caused by deletion or rearrangement [58]. Apparent chromosomal rearrangements are reflected in distinct rDNA patterns among subgenera *Strobus* (larger genomes) and *Pinus* (smaller genomes; [1], [6], [7] but not among species within each subgenus [59], which may imply distinct evolutionary processes are involved in genome size variation among *Pinus* subgenera.

There is ample precedent for periods of retrotransposition associated with species-specific genome expansion in angiosperms. A 16-fold increase in copy number of a *Gypsy* element *GORGE3* (from 5,520 in *Gossypium kirkii* to 88,492 in *G. exiguum*) occurred within the last 10 MY and, in combination with other retroelement families, account for an estimated 1,145 Mb of the total (1,872 Mb) genome size difference between these two species [60]. The *Oryza australiensis* genome has doubled within the last 3 MY, not due to polypoloidization but instead to apparently non-overlapping waves of expansion of the *Copia* element *RIRE1* and the *Gypsy* elements *Kangourou* and *Wallabi*, all of which were apparently present in the ancestor of the genus [51]. Similarly, expansion of various *Gypsy* elements has occurred within the genus *Oryza* (some by as much as 30-fold, [61]) and *Vicia* [62]. Comparative genomic sequencing in pines is required for a more precise understanding of how retrotransposon expansion has shaped genome complexity and size variation in this taxon.

Whether the evolutionary processes leading to large, complex genomes are equivalent in angiosperm and gymnosperm lineages remains an open question. Interestingly, certain classes of repeat elements show distinct chromosomal distributions in angiosperms and gymnosperms [24], [25], [63], [64] and epigenetic markings associated with heterochromatin differ in angiosperms and gymnosperms [65]. Determining whether gymnosperms share a similar distribution of elements, or exhibit a distinct genomic architecture, is a key to understanding how evolution has shaped these two major lineages of seed plants.

## Materials and Methods

### Cloning and sequence analysis

For isolation of genomic fragments adjacent to RAPD marker B8_650, *Pinus taeda* L. (genotype 10-5, obtained from NCSU Cooperative Tree Improvement Program, Raleigh, NC, USA) DNA was isolated using a CTAB based method [66], quality was checked on a 0.8% w/v agarose gel, then DNA was digested with *Dra*I, *Eco*RV, *Stu*I or *Pvu*II and ligated to adaptors according to the GenomeWalker protocol (Clontech, Mountain View, CA, USA). *Gymny* primers were designed using Netprimer (Premier Biosoft International) according to the specifications given in the GenomeWalker protocol. The GenomeWalker protocol was used for amplification of upstream and downstream regions in amplification steps using primers designed against the sequence of the B8_650 RAPD marker and adaptor primers from the GenomeWalker Kit. Gel purified PCR fragments were cloned in pGEM-T (Invitrogen, Carlsbad, CA, USA) for sequencing. Sequence assembly was done with Sequencher (Gene Codes, Ann Arbor, MI, USA) and open reading frames were identified using the ORF finder program at NCBI (http://www.ncbi.nlm.nih.gov/projects/gorf/). Sequence from a portion of a putative 5′ LTR was identified in addition to sequence containing regions similar to *pol* genes from retrotransposons. Obtaining additional sequences downstream of the integrase domain by genome walking was unsuccessful; there was an absence of optimal primer binding sites in this region, and the few amplification products obtained shared no sequence identity with the reference element. Primer sequences and amplification products obtained are listed in Figure S2.

The element was sufficiently different from other described elements, i.e., less than 80% identity over 80% of its coding regions [67], to warrant its status as the reference element of a new family. In accordance with the hierarchical nomenclature developed by Wicker *et al.* [67] the nearly complete copy of *Gymny* sequenced in our walk was designated RLG_*Gymny*_EU912388-1 based upon the class (‘R’ for retrotransposon), order (‘L’ for LTR element), superfamily (‘G’ for Gypsy), family (‘*Gymny*’ for gymnosperm), accession number (EU912388), and position with regard to other copies of the element in the accession (‘1’ for the first occurrence of *Gymny* within this accession).

The EMBOSS Isochore program was used to calculate GC content over sequence in a 100 bp sliding window (http://www.ebi.ac.uk/emboss/cpgplot/index.html). BLAST (http://www.ncbi.nlm.nih.gov) was implemented for similarity searches and SMART (http://smart.embl-heidelberg.de/) was used to search for conserved protein domains. The reverse transcriptase sequences used in the multiple-sequence comparisons in Figure 2 were obtained from GenBank (http://www.ncbi.nlm.nih.gov) and alignments generated using ClustalX [68]. A reverse transcriptase (RT) relatedness tree was assembled using ClustalX with the neighbor joining algorithm, and nodal support was assessed using 10,000 bootstrap replicates. The relatedness tree was visualized using Treeview [69].

### Fluorescent *in situ* hybridization

Chromosome spreads were prepared from root tip protoplasts of young potted *P. taeda* seedlings (progeny of genotypes LSG-62, B-5-3 and B-145-L) as described [70], [71]. Clones Fr1035 and Fr1075 (Figure 1) were labeled with biotin-16-dUTP (BIO-Nick Translation Mix, Roche Applied Science, Indianapolis, IN, USA) and 18S–28S rDNA [72] was labeled with digoxigenin-11-dUTP (Digoxigenin-Nick Translation Mix, Roche Applied Science, Indianapolis, IN, USA) following manufacturer's instructions. Hybridizations with Fr1035, Fr1075 (40 ng each per slide) and 18S–28S rDNA (25 ng per slide) were carried out as described [71] and detected with Cy3-conjugated streptavidin (Jackson ImmunoResearch, West Grove, PA, USA) or FITC-conjugated anti-digoxigenin, respectively. The hybridized chromosome spreads were counter-stained with DAPI (4 µg/ml w/v) for 5 min in the dark, washed briefly with 4× SSC/0.2% v/v Tween-20, and then mounted by Vectashield (Vector Laboratories, Burlingame, CA, USA) to prevent fluorochrome bleaching. Digital images of the hybridized and washed slides were recorded from an AxioImager Z-1 Epi-fluorescence microscope with suitable filter sets (Chroma Technology, Rockingham, VT, USA), using a COHU High Performance CCD Camera and the Metafer v4 MetaSystems Finder digital image system (MetaSystem, Belmont, MA, USA). Images were processed initially with Ikaros and ISIS v5.1 and then further processed with Adobe Photoshop CS v8 (Adobe Systems, San Jose, CA, USA).

### BAC screening

Information on the *P. taeda* (genotype 7–56) BAC library can be found at http://www.mgel.msstate.edu/dna_libs.htm. In brief, the BAC library (as of 2/18/2008) contains a total of 1,612,800 clones with a mean insert size of 94 kb and represents 7× coverage of the *P. taeda* genome. Three duplicate copies of a macroarray containing 18,432 double-spotted BAC clones were screened with overgo probes designed from the 5′ end of RLG_*Gymny*_EU912388-1 sequence. One of the probes (denoted ‘P1’, bases 5–40) corresponds to a portion of the putative 5′ LTR, a second (‘P2’, bases 609–644) comes from the region between ORF1 and the putative 5′ LTR, while the third is derived from ORF3 in the *gag* region (denoted ‘P3’, bases 2038–2073). Macroarray hybridization was performed using ^32^P-labeled overgos as described by McPherson *et al.* [73] (see http://bacpac.chori.org/overgohyb.htm for details). Briefly, hybridizations were carried out overnight at 60°C in 1 mM EDTA, 7% (w/v) SDS, 0.5 M sodium phosphate (pH 7.2) followed by a 30 minute wash at 60°C in 1 mM EDTA, 1% (w/v) SDS, 40 mM sodium phosphate (pH 7.2), a 20 minute wash at 60°c in 1.5× SSC, 0.1% (w/v) SDS and a final 20 minute wash at 60°C in 0.5× SSC, 0.1% (w/v) SDS. Hybridization images were captured using a GE Healthcare Storm 820 Phosphorimager (Piscataway, NJ, USA) according to manufacturer's instructions. Copy number estimates were obtained from representative portions of macroarrays using the protocol of Peterson *et al.* [29].

### Searching random genomic 454 reads for *Gymny*

The RLG_*Gymny*_EU912388-1 element was used as a BLASTn query against a sequence set containing 275,038 trimmed sequence reads (all reads ≥50 bases with Q≥20 over 75% of the read length; total bases = 28,039,433). The sequence set was generated by 454 pyrosequencing of random genomic DNA from the *P. taeda* genotype 7–56 (see http://www.pine.msstate.edu/seq.htm). Of the 275,038 reads, 1111 exhibited significant (bit scores>40) BLASTn hits (default parameters) to RLG_*Gymny*_EU912388-1. These 1111 reads were aligned with RLG_*Gymny*_EU912388-1 using Phrap (default parameters). The largest of the resulting Phrap contigs contained 685 of the 1111 reads and encompassed the whole RLG_*Gymny*_EU912388-1 sequence.

### Searching Cot fractionated 454 reads for *Gymny*

Highly repetitive (HR), moderately repetitive (MR), single/low-copy (SL) and theoretical single-copy (TS) Cot fractions from *P. taeda* genotype 7-56 were isolated according to Peterson et al. [29] (also see www.mgel.msstate.edu/seq_names.htm) and sequenced using a GS20 454 pyrosequencer (for sequences see www.pine.msstate.edu/seq.htm). The resulting datasets were trimmed to remove low quality sequences as described above and subjected to a BLASTn search using the RLG_*Gymny*_EU912388-1 consensus as a query, after which the top alignments with bit scores >40 were retrieved and evaluated. For comparison, we assembled each untrimmed dataset into contigs using Phrap (default parameters) and subjected the contigs to a BLASTn search using the RLG_*Gymny*_EU912388-1 consensus as a query, after which the top alignments with E values less than 1.0×10^−4^ were retrieved and evaluated. As a positive control for fractionation of genic DNA into low-copy fractions, 26 EST contigs encoding pine *WRKY* transcription factors were extracted from the Plant Transcription Factor Database (http://planttfdb.cbi.pku.edu.cn) and used as queries to interrogate the trimmed datasets. The top alignments with bit scores >40 were retrieved and evaluated.

### Southern analysis

Southern analysis was conducted using DNA isolated from foliage. In brief, 10 µg of genomic DNA were digested overnight with *Hind*III or *Hae*III enzymes at 37°C, separated (0.7% w/v agarose gel) and transferred to Hybond-N+ (Amersham Biosciences, Piscataway, NJ, USA). Probes Fr1075 and Fr1035 (Figure 1) were amplified by PCR from pGEM-T using SP6 and T7 vector primers, purified using QIAquick Gel Extraction Kit (Qiagen, Valencia, CA, USA) and labeled with radioactive ^32^P-ATP using the RadPrime DNA Labeling System (Invitrogen, Carlsbad, CA, USA). Hybridizations in aqueous buffer consisting of 0.5 M phosphate buffer, pH 7.2, 7% (w/v) SDS, 1 mM EDTA were carried out overnight at 65°C followed by a 1 hour wash in 40 mM phosphate buffer, pH 7.2, 5% (w/v) SDS, 1 mM EDTA and two stringent 30 minute washes in 40 mM phosphate buffer, pH 7.2, 1% (w/v) SDS, 1 mM EDTA at 65°C [74].

## Supporting Information

File S1 Supporting Data Analyses
(0.05 MB DOC)

Figure S1 Translated sequence alignment of Gypsy RT polymerase domains. The five RT polymerase motifs (defined by Poch et al. 1989. EMBO J. 8: 3867–3874) are indicated by black bars (A to E). Identical amino acid residues are indicated by an asterisk, conservative and semi-conservative substitutions with a colon and period, respectively. Accession numbers are Gloin AC007188.5, Gimli AL049655.2, Legolas AC006570.4, TMA AC005398, Tat4-1 AB005247.1, Tft1 AC007268.3, Tft2 AF096372, Athila1-1 AC007209.4a, Little-Athila AC007120.4, IFG7 AJ004945, PpRT1 DQ394069. All elements evaluated contain the two aspartate residues in motif C (F/YXDD) and the aspartate residue in motif A (LD) associated with RT catalytic activity.
(0.24 MB TIF)

Figure S2 GenomeWalker primers used for Gymny cloning. Primer sequences are given in (A) along with their direction relative to Gymny. Primer locations are shown in (B) within the sequences used to generate the consensus sequences.
(0.16 MB TIF)
