# Supplementary material for: Evolution of Genome Size and Complexity in Pinus
Source: PLoS One. 2009 Feb 5;4(2):e4332. doi: 10.1371/journal.pone.0004332 (PMC2633040; doi:10.1371/journal.pone.0004332)
Supplement: File S1 — Supporting Data Analyses (0.05 MB DOC) [file pone.0004332.s001.doc]

**File S1**

*Gymny* structural features

The first 500 bp of RLG_*Gymny*_EU912388-1 appears to be part of a 5’LTR based on the following characteristics; low (~30%) GC content, 3.6 tandem repeats of a 27 bp sequence and multiple small repeats (annotated in GenBank accession). The *Gymny* sequence harbors the internal sequence of a 638 bp RAPD marker linked to *Fr1* (designated B8_650; [1], but the primer annealing sites at the 5’ and 3’ termini differ from the B8 primer sequence at one and two nucleotide positions, respectively. This implies that only a subset of *Gymny* elements would be amplified by the B8 primer and the cloned element falls outside of that subset.

ORFs in *Gymny* reference element

An *in silico* sequence search for open reading frames (ORFs, NCBI ORF finder) identified a total of five ORFs, numbered 1 through 5, in reading frame 2 (Figure 1). ORF4 is interrupted by a stop codon and related to reverse transcriptases (RT) while ORF5 is related to integrase (INT) domains encoded by *pol* genes. Although sequence similarities to known *gag* regions were not detected during database searches, the locations of ORFs 1, 2 or 3 upstream of *pol* is consistent with positions of *gag* regions. An ORF3 translation revealed the presence of two modified CCHC motifs (Cys-X_3_-Cys-X_4_-Cys rather than the typical Cys-X_2_-Cys-X_4_-Cys) beginning at 1466 bp. These motifs are frequently found within the C-terminus of the nucleocapsid proteins [2] encoded by *gag*. The presence of these motifs supports the premise that this area is the *gag* region of RLG_*Gymny*_EU912388-1.

GSS and EST hits on *Gymny* reference element

To more fully delineate *Gymny* structure and to determine if *Gymny* elements are transcribed, RLG_*Gymny*_EU912388-1 was used to query the GSS (BLASTn of 5,393 entries) and EST (BLASTx of 328,628 entries) databases for *P. taeda* in GenBank (May 2008 release). Three of four GSS sequences with significant sequence identity to RLG_*Gymny*_EU912388-1 were similar to the *int* region while the fourth was similar to the putative 5’ LTR. Five ESTs with similarity to *Gymny* were identified (Table 1). One of the ESTs (NXRV099_G06_F NXRV) contains ORFs 1 and 2 corresponding to the putative *gag* region. The remaining four ESTs (EST964163, EST1156897, NXRV047_D04_F NXRV and STRR1_70_F01) are derived from the *int* catalytic domain encoded by the *pol* region.

The five *Gymny* ESTs come from three physiologically distinct tissues, suggesting that *Gymny* may be transcribed in numerous tissues under a wide variety of physiological conditions. Studies of other retrotransposons indicate that some show increased transcript abundance under stress conditions such as pathogen challenge or tissue culture [3-5] while others are transcribed under normal metabolic conditions [6,7]. The AT-rich LTRs of LTR retrotransposons contain sequences that can regulate transcription. TATA-like motifs have been tested and can function as transcription initiation elements in pea [8] and barley [9]. Although the presence of ESTs derived from *Gymny* suggest transcriptional activity of at least some members, we note that the ESTs could be a consequence of spurious transcript production from cryptic promoters or from read-through of an adjacent retrotransposon or gene.

*Gymny* similarity to *Athila* and *Little* *Athila*

*Athila* elements differ from the closely related *Little Athila* elements in that *Athila* elements contain an additional ORF3 downstream of the *pol* region that is predicted to encode a putative envelope protein [10], similar to that encoded by retroviruses [11]. Whether autonomous *Gymny* elements contain an envelope region (like *Athila*) or not (like *Little Athila*) is unknown.

*Gymny* copy number estimated by BAC overgo hybridization

These values are based on the assumption that BACs exhibiting the least intense (but clearly positive) hybridization signals contain a single copy of the probe to which they were hybridized. This may not be the case, especially with regard to the macroarray hybridized with P1. Because LTRs are typically found in pairs, the lightest positive hybridization signals may represent BACs containing two copies, which means these values would underestimate the true copy number.

*Gymny* copy number estimated by 454 hits

An independent estimate of copy number can be drawn from the percentage of *Gymny* hits from reads generated by massively parallel sequencing. In short, 1111 reads out of 275,038 (~0.40%) in the random genomic dataset aligned with RLG_*Gymny*_EU912388-1. The amount of *Gymny* DNA in the pine genome can thus be estimated at (0.0040 x 21.7 Gb =) 87,655,888 bp. If all copies of *Gymny* were similar to RLG_*Gymny*_EU912388-1, then there should be (87,655,888 bp ÷ 6200 bp =) 14,138 copies of *Gymny* in the pine genome. Although this is lower than the estimate of intact *Gymny* elements based on macroarray analysis, it is similar in magnitude.

References

1. Wilcox PL, Amerson HV, Kuhlman EG, Liu BH, OMalley DM, et al. (1996) Detection of a major gene for resistance to fusiform rust disease in loblolly pine by genomic mapping. Proceedings of the National Academy of Sciences, USA 93: 3859-3864.

2. Green LM, Berg JM (1989) A retroviral Cys-Xaa2-Cys-Xaa4-His-Xaa4-Cys peptide binds metal-ions - spectroscopic studies and a proposed 3-dimensional structure. Proceedings of the National Academy of Sciences, USA 86: 4047-4051.

3. Hirochika H, Otsuki H (1995) Extrachromosomal circular forms of the tobacco retrotransposon *Tto1*. Gene 165: 229-232.

4. Wendel JF, Wessler SR (2000) Retrotransposon-mediated genome evolution on a local ecological scale. Proceedings of the National Academy of Sciences, USA 97: 6250-6252.

5. Grandbastien MA, Audeon C, Bonnivard E, Casacuberta JM, Chalhoub B, et al. (2005) Stress activation and genomic impact of *Tnt1* retrotransposons in *Solanaceae*. Cytogenetic and Genome Research 110: 229-241.

6. Manninen I, Schulman AH (1993) *BARE-1*, a *copia*-like retroelement in barley (*Hordeum-vulgare* L). Plant Molecular Biology 22: 829-846.

7. Kalendar R, Vicient CM, Peleg O, Anamthawat-Jonsson K, Bolshoy A, et al. (2004) Large retrotransposon derivatives: Abundant, conserved but nonautonomous retroelements of barley and related genomes. Genetics 166: 1437-1450.

8. Tjaden G, Edwards JW, Coruzzi GM (1995) Cis elements and trans-acting factors affecting regulation of a nonphotosynthetic light-regulated gene for chloroplast glutamine synthetase. Plant Physiology 108: 1109-1117.

9. Cock JM, Hemon P, Cullimore JV (1992) Characterization of the gene encoding the plastid-located glutamine synthetase of *Phaseolus vulgaris*: regulation of b-glucuronidase gene fusions in transgenic tobacco. Plant Mol Biol 18: 1141-1149.

10. Marin I, Llorens C (2000) *Ty3/gypsy* retrotransposons: description of new *Arabidopsis thaliana* elements and evolutionary perspectives derived from comparative genomic data. Molecular Biology and Evolution 17: 1040-1049.

11. Wright DA, Voytas DF (2002) *Athila4* of Arabidopsis and *Calypso* of soybean define a lineage of endogenous plant retroviruses. Genome Research 12: 122-131.
