# Supplementary figures and images for: Evolution of Genome Size and Complexity in Pinus
Source: PLoS One. 2009 Feb 5;4(2):e4332. doi: 10.1371/journal.pone.0004332 (PMC2633040; doi:10.1371/journal.pone.0004332)

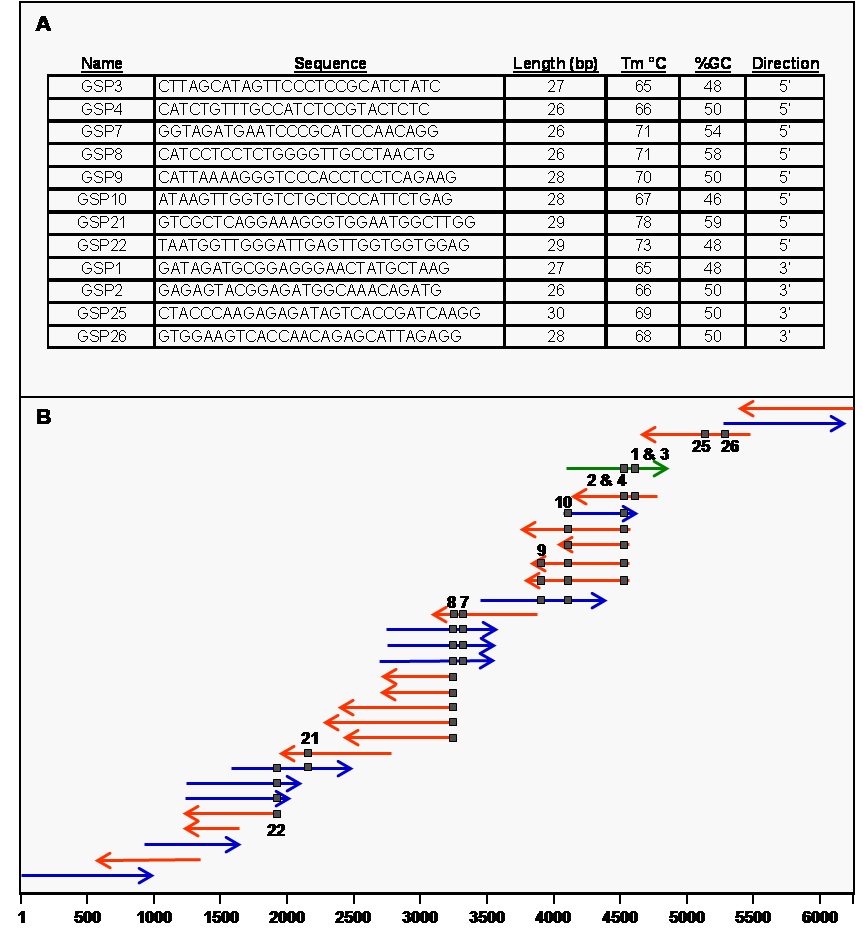

Supplement: Figure S2 — GenomeWalker primers used for Gymny cloning. Primer sequences are given in (A) along with their direction relative to Gymny. Primer locations are shown in (B) within the sequences used to generate the consensus sequences. (0.16 MB TIF) [file pone.0004332.s003.tif]
